# Phenotypic detection of flax plants based on improved Flax-YOLOv5

**DOI:** 10.3389/fpls.2024.1404772

**Published:** 2024-07-11

**Authors:** Kai Sun, Chengzhong Liu, Junying Han, Jianping Zhang, Yanni Qi

**Affiliations:** ^1^ College of Information Science and Technology, Gansu Agricultural University, Lanzhou, China; ^2^ Crop Research Institute, Gansu Academy of Agricultural Sciences, Lanzhou, China

**Keywords:** flax, YOLOv5, target detection, phenotypic data, variety breeding

## Abstract

Accurate detection and counting of flax plant organs are crucial for obtaining phenotypic data and are the cornerstone of flax variety selection and management strategies. In this study, a Flax-YOLOv5 model is proposed for obtaining flax plant phenotypic data. Based on the solid foundation of the original YOLOv5x feature extraction network, the network structure was extended to include the BiFormer module, which seamlessly integrates bi-directional encoders and converters, enabling it to focus on key features in an adaptive query manner. As a result, this improves the computational performance and efficiency of the model. In addition, we introduced the SIoU function to compute the regression loss, which effectively solves the problem of mismatch between predicted and actual frames. The flax plants grown in Lanzhou were collected to produce the training, validation, and test sets, and the detection results on the validation set showed that the average accuracy (mAP@0.5) was 99.29%. In the test set, the correlation coefficients (R) of the model’s prediction results with the manually measured number of flax fruits, plant height, main stem length, and number of main stem divisions were 99.59%, 99.53%, 99.05%, and 92.82%, respectively. This study provides a stable and reliable method for the detection and quantification of flax phenotypic characteristics. It opens up a new technical way of selecting and breeding good varieties.

## 1 Introduction

Flax (*Linum usitatissimum*) is one of the most important oil and fiber crops in the world. Flax is mainly divided into oil flax, fiber flax, and dual-purpose oil and flax varieties according to their uses (Zhang et al., 2011). Recently, the results of studies emphasizing the anticancer properties of substances present in flaxseed and oil have attracted great attention (Praczyk and Wielgusz, 2021) and are widely cultivated worldwide (Kauser et al., 2024). Selection and breeding of flax varieties are crucial for progress in flax production (Gong et al., 2020). Obtaining the phenotypic data required for flax breeding is the basis of breeding; only rapid and accurate access to flax plant phenotypic data and the breeding of flax varieties will have a qualitative leap. The traditional acquisition of flax phenotypic data is through manually counting the number of flax fruits and the number of main stems divided into stems, measuring the plant height and main stem length, and manually recording data; this traditional method of flax production has made a significant contribution to the progress of flax production, but with the advancement of science and technology, these methods have become more and more inefficient and expensive. As a result, these traditional methods often fail to meet the stringent requirements of modern breeding practices. To address these challenges, there is an urgent need to explore innovative techniques that are more efficient, cost-effective, and compatible with contemporary sub-breeding acquisition of data.

Currently, computer vision technology is widely used in agriculture and has made great progress in the accuracy and efficiency of extracting plant phenotypic data. Currently, there are two main detection methods for obtaining plant phenotypic data: traditional target detection methods and target detection methods based on deep learning (Zhang et al., 2023). Among them, the traditional target detection process is more complex, requiring multiple steps to be completed together and time-consuming, with higher requirements for images, different algorithms for different detection objects, and greater difficulty in extracting different information at the same time; deep learning has a powerful feature extraction capability, which can make up for the shortcomings of the traditional methods, and therefore, more and more researchers are using it for agricultural target detection.

In recent years, many scholars have begun to apply deep learning in the field of agriculture, such as identifying plants, pests, and diseases, to improve crop yields. Zhu et al. (2024) proposed a CBF-YOLO network for the detection of common soybean pests in complex environments. Pei et al. (2022) proposed a maize field weed detection framework based on crop row pretreatment and improved YOLOv4 in UAV images. Li et al. (2023) proposed an apple leaf disease detection method based on the improved YOLOv5s model. Bai et al. (2024) proposed an improved YOLO algorithm to detect the flowers and fruits of strawberry seedlings. Wang et al. (2024) developed a new deep learning network, YOLO-DCAM, which effectively facilitates single-wood detection in complex scenarios. Du et al. (2023) proposed a method for detecting strawberry fruit planted in fields under different shade levels. Su et al. (2023) proposed an improved YOLOv5-SE-BiFPN model, which could more effectively detect brown spot lesion areas in kidney beans. Zhang et al. (2024) proposed a multi-task learning method named YOLOMS for mango recognition and rapid location of major picking points.

YOLO series is a single-stage algorithm that ensures high precision and faster speed, especially in the GPU environment, and real-time detection can be realized. Due to its excellent performance, it has achieved great results in the extraction of plant phenotype data and the application of detection objects. Guo et al. (2022) proposed a method to obtain phenotypic parameters of soybean plants based on Re-YOLOv5 and detection region search algorithms, and the results showed that the average absolute errors of plant height, stem node count, and soybean branch count were 2.06 cm, 1.37 cm, and 0.03 cm, respectively. The results were better, and a specialized black box for filming was developed, but this is time-consuming in the face of a large number of films to obtain phenotypic data and does not apply to realistic breeding requirements. Chen et al. (2024) proposed an efficient, fast, and real-time seedling counting method for cabbages, which replaced the C2f block in the main stem network of YOLOv8n with a Swin-conv block and added a ParNet block to both the main stem and neck portions of the network. ParNet attention modules were added to the neck section to accurately track cabbage seedlings in the field and count them using an unmanned aerial vehicle (UAV), achieving 90.3% mAP50–95, but its recognition progress needs to be further improved. She et al. (2022) introduced the ECA attention mechanism into the YOLOv5s model to improve the accuracy of trap vial detection and counting, but the recognition accuracy needs to be further improved. Gao et al. (2022) proposed the YOLOv4-tiny network combined with the channel spatial reliability discriminant correlation filtering (CSR-DCF) algorithm for training, and the correlation coefficient R^2^ between apple number prediction and manual counting was 0.9875. The counting accuracy of the orchard video is 91.49%, so the accuracy of fruit recognition in the video needs to be further improved.

While deep learning has applications in acquiring plant phenotypic data, it has received limited attention for the accurate detection of organs in flax plants. In real-world detection scenarios, complex flax fruit overlap and branching pose significant challenges to fruit occlusion. This often leads to incomplete detection, as existing models ignore occluded flax fruits. In addition, less characterization of flax plant main stem length and main stem branching increases the complexity of identification. In addition, the shapes of flax fruits, plant heights, industrial lengths, and main stem meristems varied, increasing the difficulty of designing a fusion model for identification. To solve these problems and improve the accuracy of phenotypic information, this study proposes a pioneering method to recognize phenotypic organs of flax plants, and this technological breakthrough is expected to improve the efficiency of breeding and open up a new way for precision agriculture. The main contributions are summarized as follows.

(1) Establishing a new flax plant dataset.
(2) Deepening the original YOLOv5x network layer and adding the BiFormer attention mechanism to its network layer significantly improve the extraction of flax features and reduce the risk of overfitting (Yang et al., 2023). In addition, the SIoU loss function replaces the original CIoU loss function, which effectively solves the problem of mismatch between the prediction and the actual bounding box and improves the accuracy of the model (Qian et al., 2024).
(3) After the model is fully trained, it is loaded onto the test set for identification and compared with the manual test data to obtain a good correlation. The model has been embedded into PC software and put into use.

The rest of the paper is organized as follows. Section 2 discusses the methods involved in the flax plant dataset, the improved Flax-ylolv5, the experimental setup, and the evaluation criteria. The conclusions are explained and discussed in Section 3. The design of the improved Flax-YOLOv5 application software is presented in Section 4. Section 5 summarizes the conclusions of the paper.

## 2 Materials and methods

### 2.1 Phenotypic dataset of flax plants

The experimental study used manually collected samples of mature and intact plants of flax from the Lanzhou Flax Planting Base of Gansu Provincial Academy of Agricultural Sciences. A total of 630 flax plants were collected to ensure phenotypic diversity. These samples were carefully selected to include a range of plant types, such as single main-stem split-stem flax plants, multiple main stem split-stem flax plants, flax plants with different numbers of fruits, and plants with complex branching patterns.

Images were captured using an MV-HS2000GM/C2 industrial camera. To eliminate potential interference from natural light, which can lead to exposure problems and complex backgrounds, the shoot was conducted indoors. A LED light source was used to provide supplemental lighting during the shoot, while a black light-absorbing cloth was used as a backdrop to simplify the test background and minimize interference. Additionally, the branches of the flax plants were hand-arranged to prevent excessive fruit overlap. To ensure accurate measurement of plant height and main stem length, the flax plant was placed horizontally below the camera lens. The camera height was set to 140 cm, and the image resolution was set to 5,472 pixels × 3,000 pixels to capture high-quality images for subsequent analysis.

### 2.2 Labeling of phenotypic feature datasets

The image features obtained were carefully measured and annotated for specific phenotypic traits, including the number of flax fruits, plant height, length of the main stem, and number of divisions within the main stem. Length measurements were made in centimeters with accuracy maintained to one decimal place. Considering the irregularity of traits such as number of flax fruits, plant height, length of the main stem, and branching of the main stem, we aimed to minimize measurement errors. Therefore, all phenotypic traits of flax plants were labeled to represent the average of three separate measurements. The labeling process utilized a dedicated labeling tool to generate the dataset in text format. The number of fruits on the flax plant, recorded as complete fruits, was labeled as “flax”. Plant height, which represents the vertical extension of the plant from root to tip, was labeled as “height”. The length of the main stem, i.e., the distance from the root to the first main branch, is labeled as “length”. In addition, the number of divisions, representing the number of branches emanating from the prominent main stem, was labeled “n” (n = 1, 2, …), and the maximum number of main stem divisions observed in a single plant was six.

### 2.3 Data expansion

**Figure 1:**
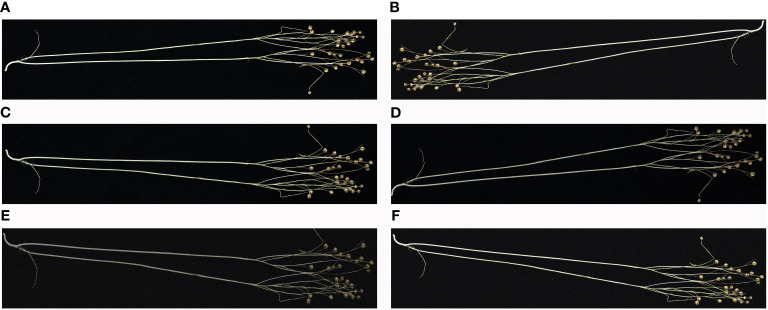
Example of data enhancement: **(A)** original, **(B)** rotated, **(C)** mirrored, **(D)** reduced brightness, **(E)** mirrored and reduced brightness, and **(F)** mirrored and added noise. The image has been cropped for ease of viewing.

A traditional data enhancement method was used to enrich the diversity of flax plant image samples, thus enhancing the generalization ability and robustness of the model. The enhancement process was carried out in five different ways: downward brightness adjustment, mirror operation, rotating the image, a combination of mirroring and brightness reduction, and a combination of mirroring and noise addition. **Figure 1** shows an illustrative example of this data enhancement process, which demonstrates the effectiveness of these techniques in generating a diverse and representative sample of images to be used for model training.

### 2.4 Original YOLOv5x

**Figure 2:**
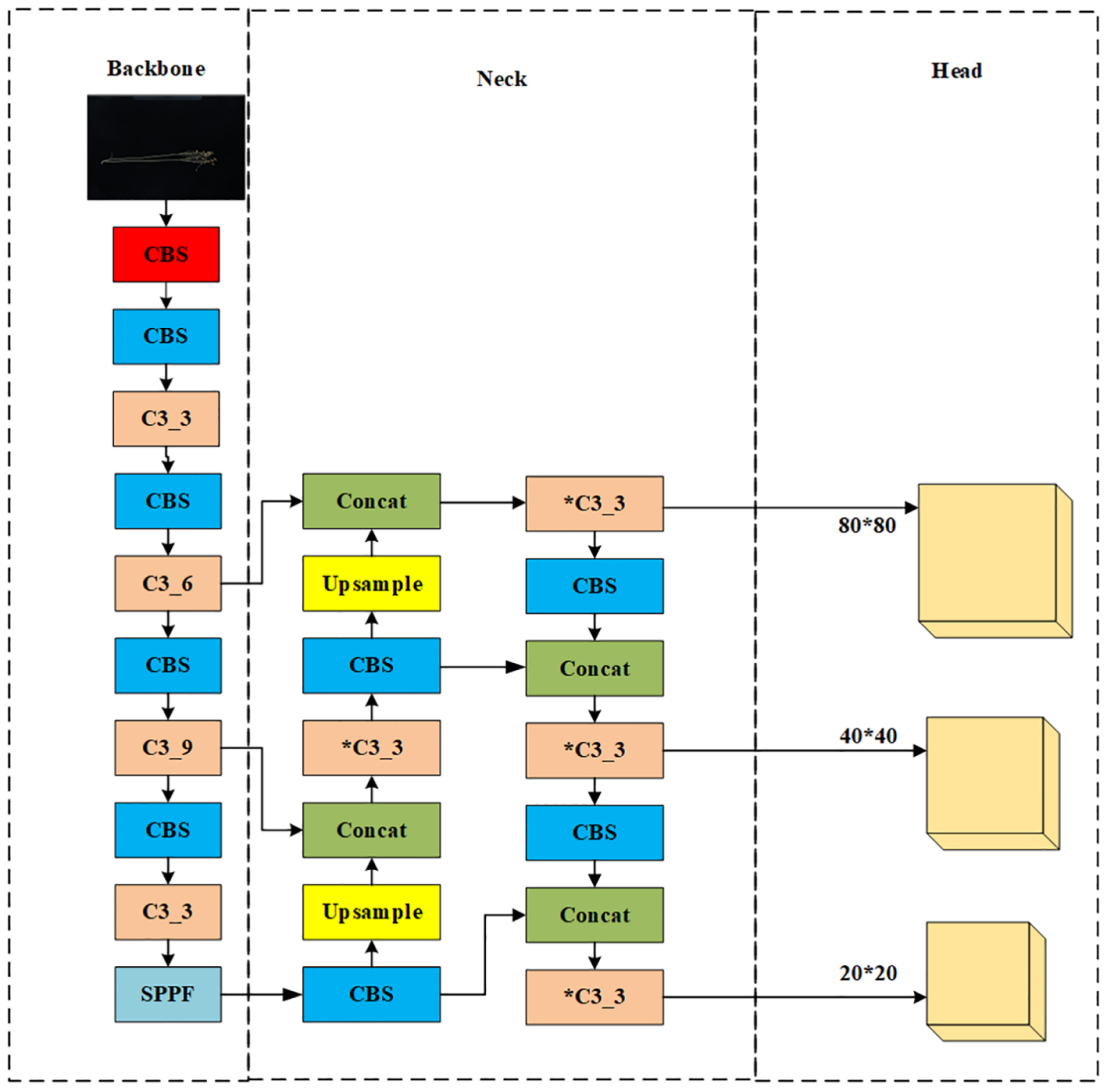
YOLOv5x model structure.

**Figure 3:**
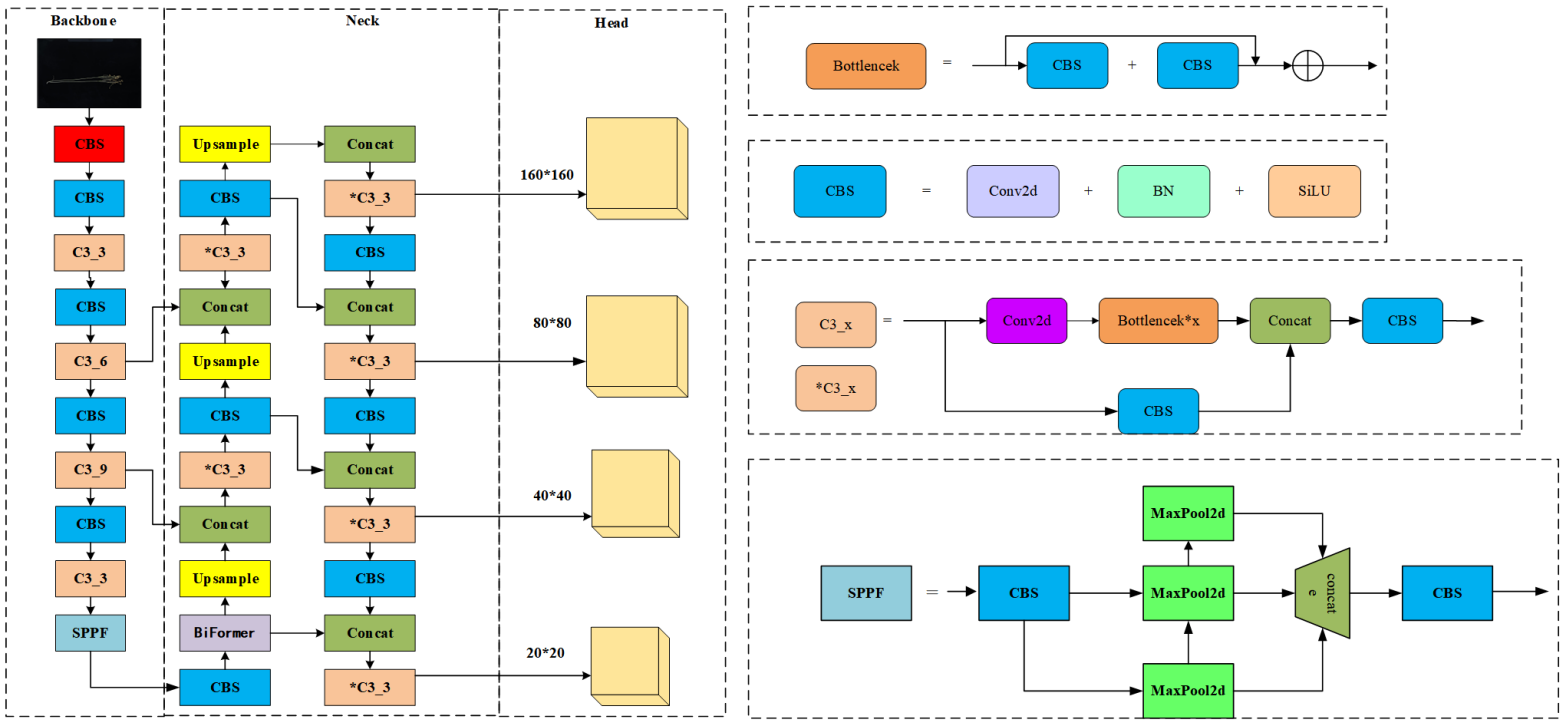
Flax-YOLOv5 model structure.

As shown in **Figure 2**, the original network structure of YOLOv5x is divided into an input network, a backbone network, a neck network, and a head network. The input integrates mosaic data enhancement, adaptive anchoring, and adaptive image scaling of 1.33 depth and 1.25 width. The backbone is a convolutional neural network that accumulates fine-grained images and generates feature maps. It contains CBS, C3, and Spatial Pyramid Pooling (SPPF) for feature extraction as shown in **Figure 3**. The YOLOv5x neck part uses a PANet structure for multi-scale feature fusion. The neck network combines the feature maps collected by the backbone network and then passes the integrated feature maps to the head network, which generates predictions from the anchor box for target detection (Rahman et al., 2022). The head network outputs a vector containing the class probability of the target, the target score, and the location of the bounding box around the target.

### 2.5 Improved Flax-YOLOv5

To accurately identify the phenotypic organs of flax plants, a Flax-YOLOv5 network structure model with high detection accuracy and detection speed was proposed. First, in the Flax-YOLOv5 network shown in **Figure 3**, the adaptive image scaling of Flax-YOLOv5 is 1.0 times depth and 1.0 times width. This adjusts the depth and width of the network to meet the needs of different scenes and improve detection accuracy.

Second, the Flax-YOLOv5 backbone network is improved based on the inheritance of the YOLOv5x backbone network. In the improvement of Flax-YOLOv5, the BiFormer module is added after the CBS module at layer 10 in the original YOLOv5x necking network. The CBS module, Upsample, Concat, and C3 modules are added at the end of the 18th layer, and the CBS, Concat, and C3 layers are added at the end of the 28th layer to improve the model’s ability to extract target features.

Finally, the improved Flax-YOLOv5 head network in **Figure 3** generates feature maps with sizes of 160 × 160, 80 × 80, 40 × 40, and 20 × 20 with different scale target detection; the improved network model is named Flax-YOLOv5, and its structure is shown in **Figure 3**.

Flax-YOLOv5 is divided into three parts. The backbone is used for feature extraction of input Flax plant images, the Neck is used for feature fusion of acquired feature mappings, and the Head is used for regression prediction. BiFormer is introduced into the feature fusion network Neck to improve the feature extraction capability of the model. Second, the SIoU function is introduced into the output Head to calculate the regression loss and improve the convergence ability of the model. Among them, the CBS module is a basic convolutional neural network module, used to extract and transmit image features; it is composed of Conv (CONvolution layer), BN (Batch Normalization layer), and SiLU (activation function) in three parts. The Conv layer is responsible for the convolution operation of the input feature graph to extract higher-level features. The BN layer is used to normalize the data, which helps accelerate training and improve the performance of the model. SiLU (Sigmoid-weighted Linear Unit) is an activation function to increase the non-linearity of the model. The C3_x module is composed of a series of multiple residual network structures. The inner Bottleneck module can be programmed to divide C3_x into two different structures, which are applied in the Backbone network and Neck network. The outer layer of the C3_x module connects to the CBS module to form a large residual edge. These residual components enhance the feature extraction capability of convolutional networks, and the stacking of residual blocks solves the difficult balance between network depth and gradient. C3_3 indicates that the C3 module has three Bottleneck modules. The SPPF module is an improved version of the Spatial Pyramid Pooling (SPP) module. SPP module is mainly used for image recognition and target detection, which can extract and encode image features at different scales, re-scale input images of any size to a fixed size, and generate fixed-length feature vectors. The SPPF module changes the parallel structure of SPP to a serial structure, which significantly reduces the amount of computation and makes the speed faster. This improvement not only maintains the function of SPP but also significantly improves the speed.

#### 2.5.1 BiFormer attention mechanism

**Figure 4:**
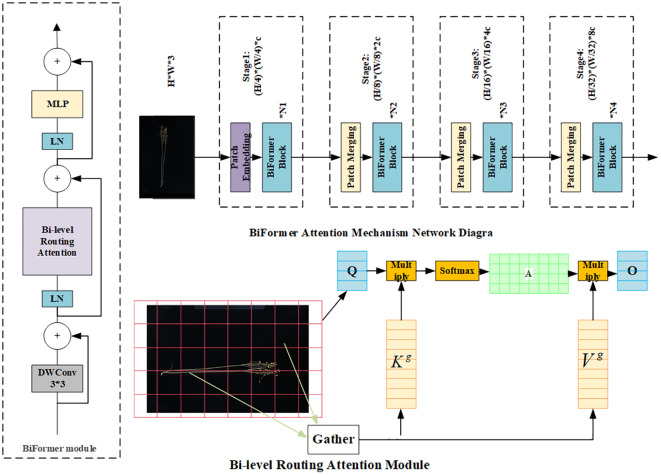
BiFormer attention mechanism architecture.

In the original image, the flax fruit is a small target with fewer features in terms of main stem length and number of main stem branches. For better extraction of effective features, the BiFormer module is introduced. BiFormer focuses on a small number of relevant markers in a query-adaptive manner without distracting other irrelevant markers, thus providing good performance and high computational efficiency. BiFormer is used in the first stage using overlapping block embedding, and in the second stage through the fourth stage, it uses a block merging module to reduce the input spatial resolution while increasing the number of channels and then uses consecutive BiFormer blocks for feature transformation. Note that the relative position information is implicitly encoded at the beginning of each block using 3 × 3 deep convolution. Subsequently, the (Bi-level routing attention, BRA) module and the 2-layer Multi-Layer Perceptron (MLP) module with an expansion rate of e are sequentially applied for cross-positional relation modeling and position-by-position embedding, with the BiFormer attention mechanism shown in **Figure 4** (Kong et al., 2023).

#### 2.5.2 SIoU

YOLOv5x uses the CIoU loss function, which is a traditional loss function for target detection that relies on the aggregation of bounding box regression metrics and does not take into account the desired orientation mismatch between the real and predicted frames, resulting in slower convergence and lower efficiency. To solve this problem, the loss function SIoU is introduced in the improved model, which considers not only the overlap region, distance, and orientation but also the angle between the predicted frame and the true frame. The SIoU formula is defined by Equations 1–5, where IoU is the regular regression loss, Δ is the distance loss, Ω is the shape loss, *B* denotes the prediction frame, *B^gt^* denotes the ground truth box, *ω^gt^* and *h^gt^* denote the width and height of the ground truth box, respectively, and ω and ℎ denote the width and height of the prediction box. b and *b^gt^* denote the centroid of the predicted truth box and the true truth box, respectively, and $b_{cx}^{gt}$ and $b_{cy}^{gt}$ denote the horizontal and vertical coordinates of the center of the ground truth box, respectively. *b_cx_* and *b_cy_* are the corresponding coordinates of the predicted box. *θ* is an adjustable parameter used to control how much to focus on the shape cost, which is set to 4 in this study (Zhang et al., 2024).


(1) $${\text{Loss}}_{SIoU}=1-IoU+\frac{\Delta +\Omega }{2}$$

(2) $$\begin{array}{c} Iou=\frac{B\cap B^{gt}}{B\cup B^{gt}},\beta =\frac{\upsilon }{\left(1-IOU\right)+\upsilon },\\ \upsilon =\frac{4}{{\text{π}}^{2}}{\left(tan^{-1}\frac{\omega^{gt}}{h^{gt}}-tan^{-1}\frac{\omega }{h}\right)}^{2} \end{array}$$

(3) $$\begin{array}{c} \Delta =\sum_{t=x,y}(1-e^{-\gamma \rho t}),\rho_{x}=\frac{b_{cx}^{gt}-b_{cx}}{c_{w}},\\ \rho_{x}=\frac{b_{cy}^{gt}-b_{cy}}{c_{h}},\gamma =2-\Lambda \end{array}$$

(4) $$\text{Λ}=1-2\;*\;sin^{2}(\arcsin\left(x\right)-\frac{\pi }{4},x=\frac{c_{h}}{\sigma }=sin\alpha$$

(5) $$\text{Ω}=\sum_{t=\omega ,h}{\left(1-e^{-\omega_{t}}\right)}^{\theta },\omega_{\omega }=\frac{\left|\omega -\omega^{gt}\right|}{\text{max}\left(\omega ,\omega^{gt}\right)},\omega_{h}=\frac{\left|h-h^{gt}\right|}{\text{max}\left(h,h^{gt}\right)}$$

## 3 Improved model identification results and analysis

### 3.1 Experimental process

**Figure 5:**
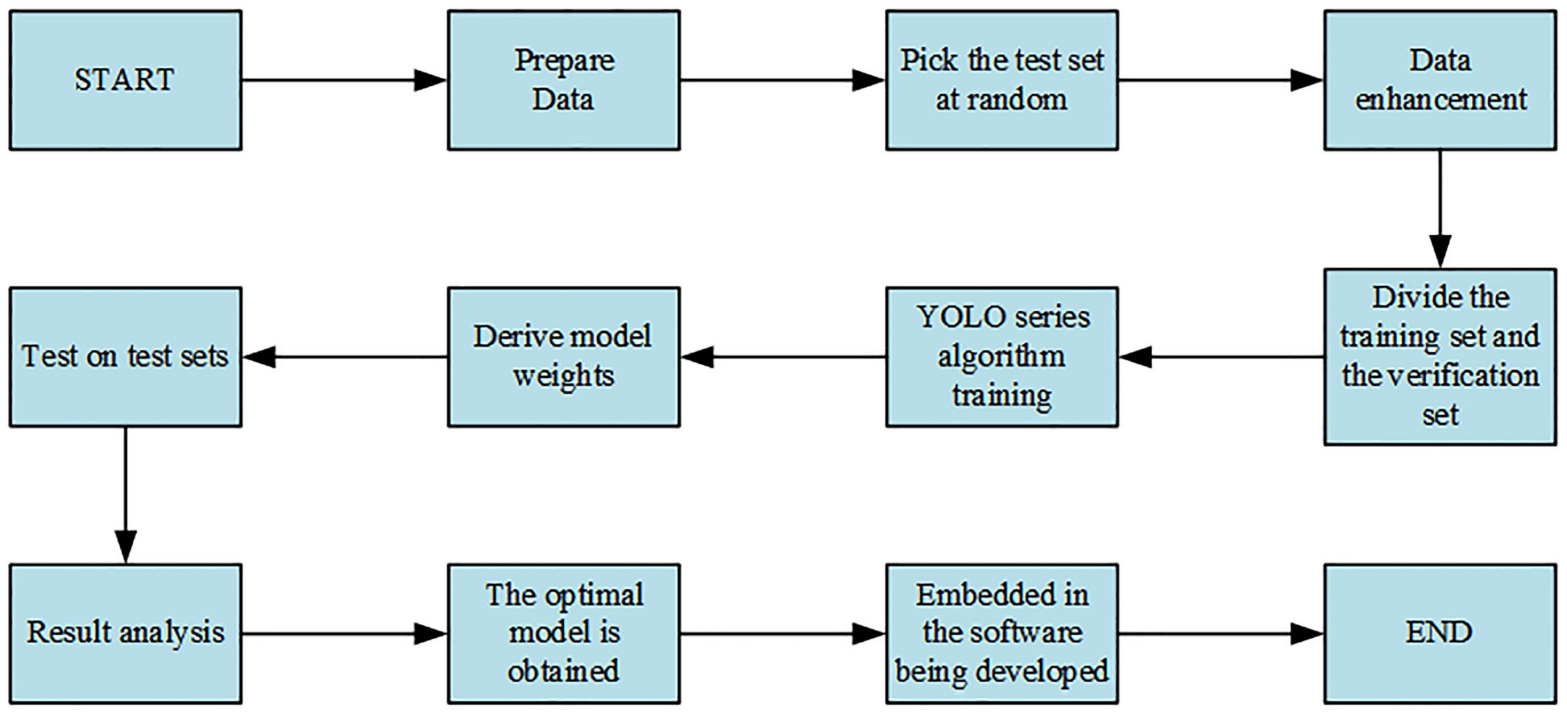
Experimental flowchart.

The specific steps of the experiment are shown in **Figure 5**.

As shown in **Figure 5**, data collection was carried out first. Of the 630 images collected, 100 were selected as the test set, and the remaining 530 images, that is, 3,180 images obtained through five data enhancement methods, were randomly divided into the training set and the verification set according to the ratio of 8:2, among which 2,544 were the training set. The verification set was 636 pieces. Second, the YOLO series model was trained on the training set. Finally, the model weight obtained from the above model on the training set was loaded onto the corresponding model and then tested on the test set. The optimal model was obtained by comparing the obtained results, and the optimal model was embedded in the developed software for the convenience of flax breeders.

### 3.2 Experimental environment

**Figure 6:**
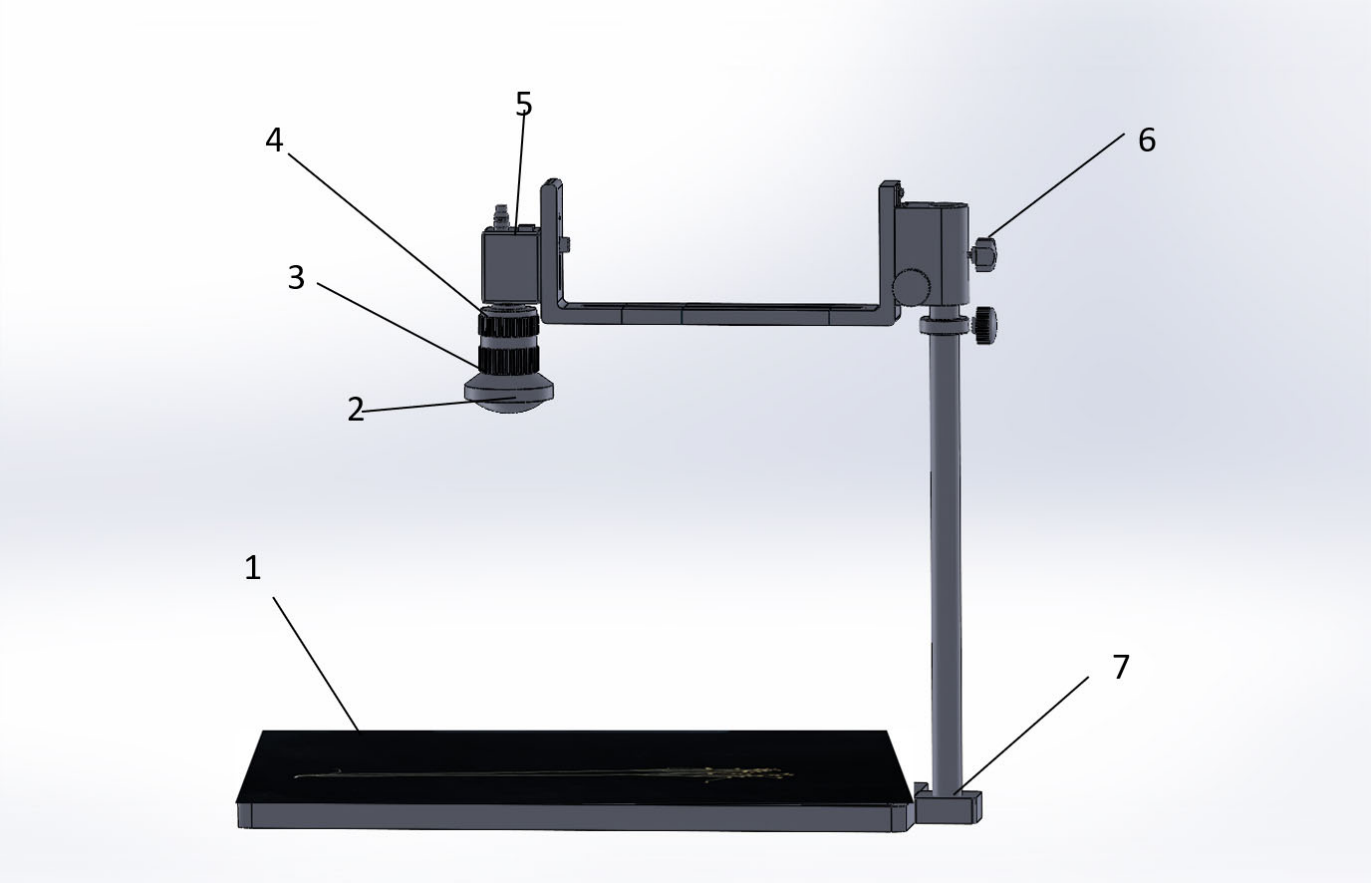
Shooting instrument. (1) Flax plant carrier table, (2) industrial camera wide-angle lens, (3) exposure time adjustment, (4) focal length adjustment, (5) computer data cable connection, (6) height adjustment, and (7) removable metal tube.

All models completed training on a server configured with CPU: Intel^®^ Xeon^®^ W-2123 CPU @ 3.60GHz and GPU: RTX 1080Ti with 8-GB video memory. The model training environments were PyTorch 1.10.0, python 3.8, and Cuda 10.2. The training parameters were 300 epochs (Ajayi et al., 2023); batch size was 4; the learning rate was set to 0.01, 0.937 momentum, 0.0005 weight decay, 0.2 IoU, 0.015 hue, 0.7 saturation, 0.4 lightness, 1.0 mosaic, 0.5 scale, and 0.1 translate; image input resolution was 640 pixels × 640 pixels; other original default parameters were used. The shooting instrument is shown in **Figure 6**.

### 3.3 Evaluation metrics

In this study, in addition to using the target detection algorithm to evaluate the precision and recall metrics, as well as the metrics for F1, we evaluated the Mean Average Precision (mAP) performance of the model at an Intersection over Union (IoU) threshold of 0.5. In addition, to assess the accuracy of the phenotypic parameters extracted from flax plants using the Flax-YOLOv5 model, four evaluation metrics were used: mean absolute error (MAE), maximum absolute error (HAE), root mean square error (RMSE), and correlation coefficient (R). The above evaluation metrics can be defined by Equations 6–15. tP is true positive (correctly detected), FN is false negative (not detected), FP is false positive (incorrectly detected), F1 is the trade-off between precision and recall, mAP is the average of all the AP values of the different categories, MAE is the average of all the absolute errors, and HAE is the maximum absolute error. RMSE is very sensitive to the magnitude error of a set of measurements and gives a good indication of the precision of the measurements. r is the degree of correlation between the manually measured flax plant phenotypic data and the model-predicted data, N is the number of experimental images, Ti is the manually measured ith plant phenotypic data, and mi is the model-predicted ith plant phenotypic data. These metrics were chosen to comprehensively evaluate the phenotypic data extraction ability of the directed search algorithm (Abyaneh et al., 2011).


(6) $$Precision=\frac{TP}{TP+FP}\times 100\%$$

(7) $$Recall=\frac{TP}{TP+FN}\times 100\%$$

(8) $$F=\frac{{\left(\alpha^{2}+1\right)}^{2}Recall\times Precision}{Recall+\mathrm{Pr}ecision}$$

(9) $$F1=\frac{2\;*Recall\times Precision}{Recall+Precision}$$

(10) $$AP=\int_{0}^{1}Precision_{\left(Recall\right)}dR$$

(11) $$mAP=\frac{\sum_{i=1}^{N}AP_{i}}{N}$$

(12) $$MAE=\frac{1}{N}\sum_{1}^{N}\left|m_{i}-T_{i}\right|$$

(13) HAE=Max(|mi−Ti|)

(14) $$\begin{array}{l} RMSE=\sqrt{\frac{1}{\text{N}}\sum_{1}^{\text{N}}{\left(m_{\text{i}}-T_{i}\right)}^{2}} \end{array}$$

(15) $$R=\sqrt{1-\frac{\sum_{i=1}^{N}{\left(m_{i}-T_{i}\right)}^{2}}{\sum_{i=1}^{N}{\left(m_{\text{i}}-\bar{m}\right)}^{2}}}$$

### 3.4 Calculate the number of flax fruits, plant height, length of main stem, and number of main stem divisions

(1) Number of flax fruits

The number of flax fruits is determined by the number of “Flax” labels.

(2) Plant height and main stem length

In the same environment, Formulas 16 and 17 define the flax plant height and main stem length: H_true_ is the manually measured value of plant height and main stem length of the flax plant, H_pi_ is the plant height and main stem length of the pixel of the identification frame, H_rate_ is the ratio of the actual length of the one-dollar coin to the length of the pixel, H_rate2_ is the actual length of the one-dollar coin, and H_pi2_ is the pixel length of the one-dollar coin.


(16) $$H_{true}=H_{pi}\;*\;H_{rate}$$

(17) $$H_{rate}=\frac{H_{true2}}{H_{pi2}}$$

The actual diameter of the one-dollar coin was measured using 0.02-mm Vernier calipers, and the pixel diameter of the one-dollar coin was calculated using digital image technology.

(3) Number of main stem divisions

The label “n” (n= 1, 2, …) indicates that the main stem of the flax plant is n sub-stems, from which the number of sub-stems of the main stem is calculated.

### 3.5 Model identification results

**Figure 7:**
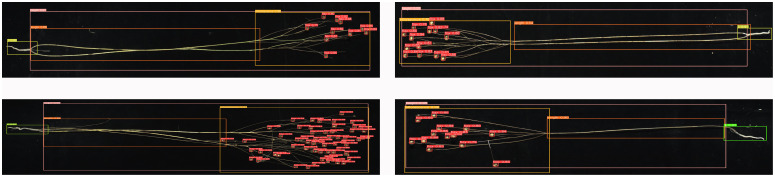
Results of phenotypic organ recognition in flax plants. The image has been cropped for ease of viewing.

**Figure 8:**
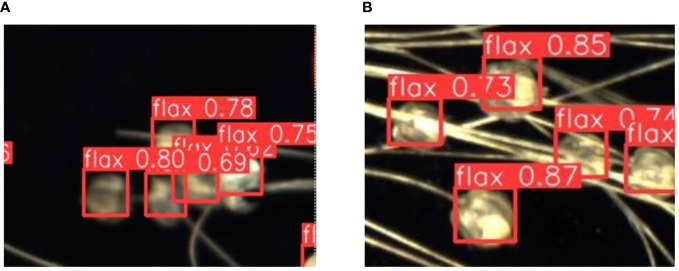
Recognition results of partially obscured fruits. The label “flax” in the picture stands for flax fruit; Numbers are confidence rates. **(A)** demonstrates the case of some flax fruits occluding each other, while **(B)** demonstrates the case of branches occluding flax fruits.

The phenotypic organs of 100 flax plant images from the test set were recognized using the improved Flax-YOLOv5 model. The results of flax plant phenotypic organ recognition are shown in **Figure 7**. In addition, **Figure 8A** demonstrates the case of some flax fruits occluding each other, while **Figure 8B** demonstrates the case of branches occluding flax fruits, from which it can be seen that the model proposed in this paper has better recognition results.

**Figure 9:**
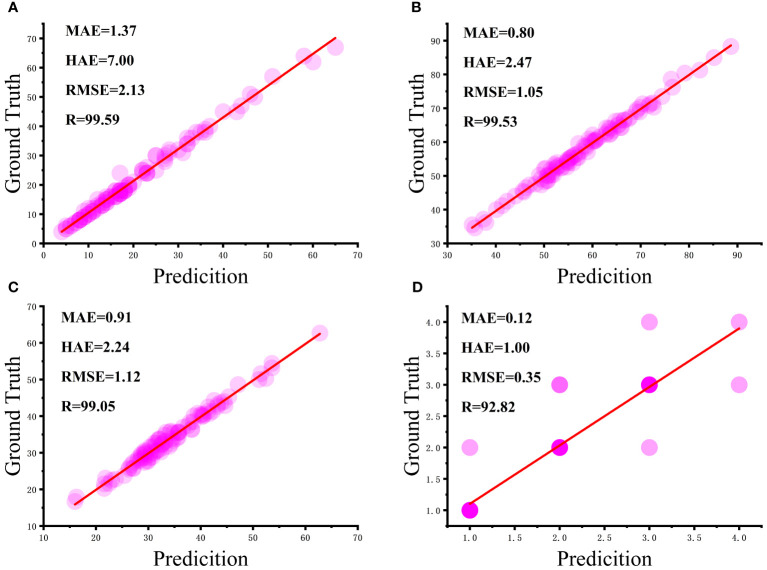
Correlation analysis between manual and algorithmic measurements: **(A)** number of flax fruits, **(B)** plant height, **(C)** length of main stem, and **(D)** number of main stem divisions.

The phenotypic data of 100 flax plants obtained from manual measurements were thoroughly compared with the phenotypic prediction data generated by the algorithm proposed in this study. To assess the reliability and stability of the algorithm in this paper, a correlation analysis was performed, and the results are shown in **Figure 9**.

From **Figure 9A**, it can be seen that most of the flax plants had between five and 40 fruits with a strong correlation and a mean absolute error of 1.37 fruits, although the maximum absolute error was seven fruits, but this was for very few plants with complex branching. As can be seen in **Figure 9B**, the height of most plants ranged from 50 cm to 75 cm, with a mean absolute error of 0.80 cm. As can be seen in **Figure 9C**, the craft length of the majority of plants was essentially in the range of 30 cm to 50 cm, with a mean absolute error of 2.24 cm. It is worth noting in **Figure 9D** that the intensity of the bubble color in the graphs reflects the number of main stem divisions of the repeat frequency, the vast majority of the main stem split number predicted accurately, with an average absolute error of 0.12. In summary, the number of fruits, plant height, main stem length, and the number of main stem split R of flax plants was 99.59%, 99.53%, 99.05%, and 92.82%, respectively, and the results were better and in line with the actual production needs.

### 3.6 Validation set test results and analysis

**Figure 10:**
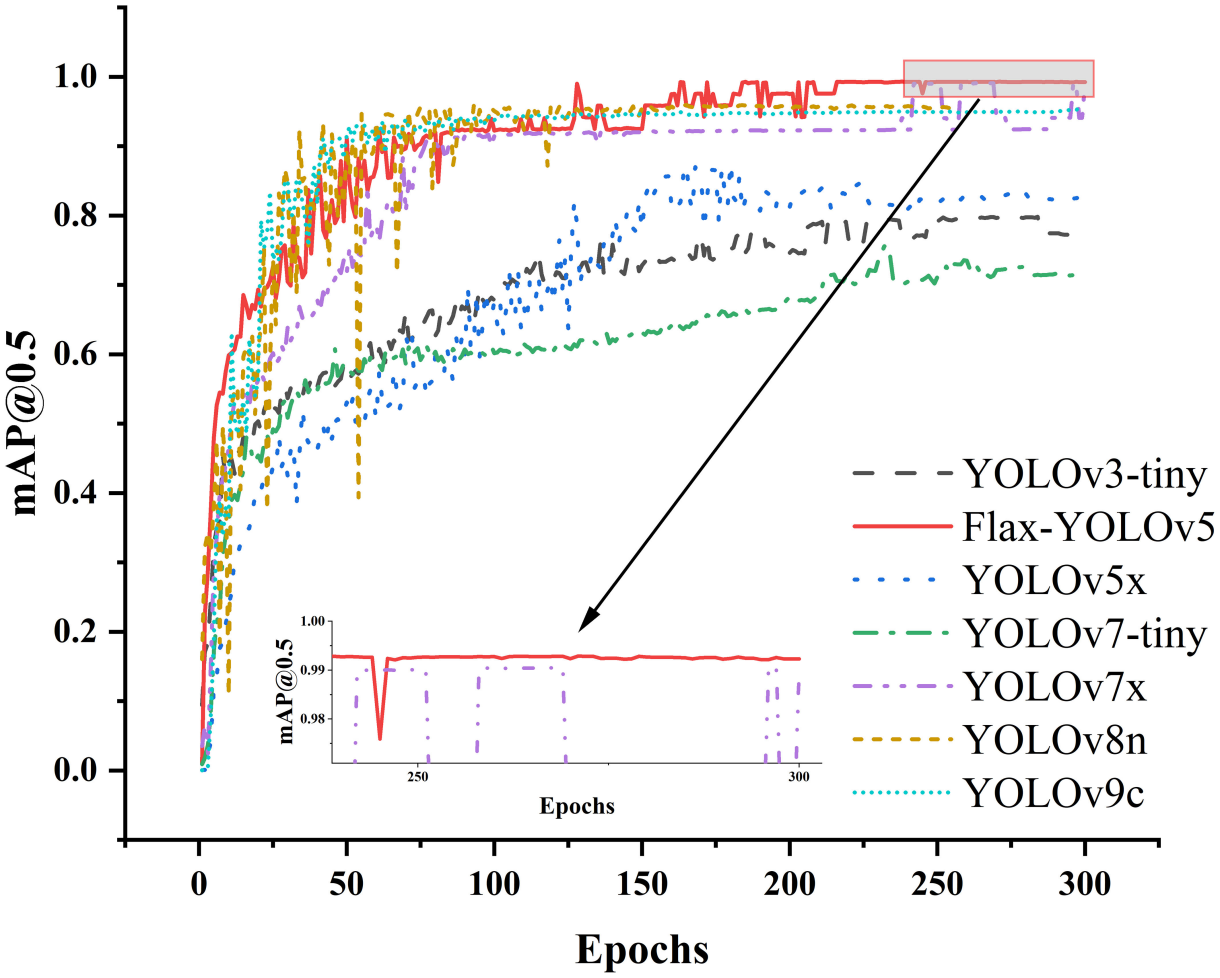
mAP@0.5 change curves of different models.

To evaluate the performance of the Flax-YOLOv5 model, we performed tests on a validation set. We chose the YOLOv3-tiny (Redmon and Farhadi, 2018), YOLOv5x (Jocher et al., 2022), YOLOv7-tiny (Wang et al., 2023), YOLOv7x, YOLOv8n (Lou et al., 2023), and YOLOv9c (Wang et al., 2024) models for comparison. Changes in training curves of different models mAP@0.5 are shown in **Figure 10**. It can be seen from the figure that mAP@0.5 of the YOLOv3, YOLOv5x, YOLOv7-tiny, YOLOv8n, and YOLOv9c models is significantly lower than that of the improved model Flax-YOLOv5. Although mAP@0.5 of the YOLOv7x model is close to that of the Flax-YOLOv5 model, it does not exceed it, and mAP@0.5 of the Flax-YOLOv5 model tends to 1 in a more stable trend with stronger convergence.

The experimental results comparing the recognition accuracy of the improved model Flax-YOLOv5 model with other models are shown in **Table 1**. As can be seen from **Table 2**, F1 and mAP@0.5 values of YOLOv3, YOLOv5x, and YOLOv7-tiny models are lower than 90%, which indicates that the performance is not ideal and does not meet the requirements of actual applications. Compared with the YOLOv7x model, the Flax-YOLOv5 model has an increase of 0.56 percentage points on F1 and 0.22 percentage points on mAP@0.5. However, the Flax-YOLOv5 model is 36.22 MB less than the YOLOv7x model. Although the YOLOv8n and YOLOv9c models are smaller than the improved model, the F1 evaluation shows that the improved model has more advantages. Overall, the improved Flax-YOLOv5 model exhibits superior performance compared to the YOLOv3, YOLOv5x, YOLOv7-tiny, YOLOv7x, YOLOv8n, and YOLOv9c models, providing a balance between accuracy and model size.

**Table 1:** Results predicted by different models in the test set.

Model	Number of flax fruits/pieces				Plant height/cm				Main stem length/cm				Number of main stem divisions/pieces			
	MAE	HAE	RMSE	R	MAE	HAE	RMSE	R	MAE	HAE	RMSE	R	MAE	HAE	RMSE	R
YOLOv3-tiny	21.16	67.00	25.18	7.69	/	/	/	/	/	/	/	/	/	/	/	/
YOLOv5x	18.76	61.00	23.00	26.76	2.01	5.84	2.51	97.91	8.27	54.40	14.65	37.41	1.58	4.00	1.92	12.85
YOLOv7-tiny	9.37	39.00	12.87	89.03	1.40	5.57	1.78	99.04	5.60	51.60	12.21	45.40	1.28	4.00	1.74	19.24
YOLOv7x	5.97	24.00	8.60	94.55	1.28	6.22	1.60	98.94	4.40	42.90	9.99	63.78	0.32	4.00	0.73	70.47
YOLOv8n	19.14	62.00	23.01	53.94	2.01	23.60	4.06	92.76	6.59	51.60	13.65	38.71	0.55	4.00	1.07	48.56
YOLOv9c	19.43	60.00	23.01	72.41	1.21	4.86	1.55	99.15	3.74	44.3	9.25	66.00	0.34	3.00	0.72	74.55
Flax-YOLOv5	1.37	7.00	2.13	99.59	0.80	2.47	1.05	99.53	0.91	2.24	1.12	99.05	0.12	1.00	0.35	92.82

MAE, mean absolute error; HAE, maximum absolute error; RMSE, root mean square error; R, correlation coefficient.

**Table 2:** Comparison of recognition results of different models.

Model	Precision (%)	Recall (%)	F1 (%)	mAP@0.5 (%)	Model size (MB)
YOLOv3-tiny	81.90	75.92	78.80	79.73	17.15
YOLOv5x	88.01	62.68	73.22	87.60	169.22
YOLOv7-tiny	92.61	66.31	77.28	71.26	12.03
YOLOv7x	92.82	98.15	95.41	99.07	138.88
YOLOv8n	94.58	91.31	92.92	95.75	6.14
YOLOv9c	95.51	90.77	93.08	95.35	50.44
Flax-YOLOv5	93.25	98.86	95.97	99.29	102.66

### 3.7 Test set test results and analysis

In this study, four phenotypic data points for each flax plant sample corresponding to the images in the dataset were successfully obtained through rigorous testing of the test set. These phenotypic measurements were then compared with manual measurements for validation. The results predicted by the different models in the test set are given in **Table 1**.

The YOLOv3-tiny model showed limited discrimination, recognizing only the fruits of the flax plant with a correlation coefficient of only 7.69%, indicating a large margin of error. Similarly, the identification results of the YOLOv5x model showed correlation coefficients of less than 50% for the number of flax fruits, main stem length, and number of main stem meristems, reflecting considerable inaccuracy.

The YOLOv7-tiny, YOLOv8n, and YOLOv9c models also performed poorly in the identification of flax fruit number, main stem length, and main stem branching number. The correlation coefficient of the YOLOv7x model in identifying the main stem length and the main stem branching number was less than 50%, and the identification accuracy was poor, with correlation coefficients of identifying the main stem length and the main stem branching number being 63.78% and 70.04%, which were unsatisfactory.

The improved Flax-YOLOv5 model, in contrast, showed better prediction results, with correlation coefficients of 99.59%, 99.53%, 99.05%, and 92.82% for flax fruit, plant height, main stem length, and number of main stem branches, respectively. These results were significantly better than those of the YOLOv3-tiny, YOLOv5x, YOLOv7-tiny, YOLOv7x, YOLOv8n, and YOLOv9c models.

**Figure 11:**
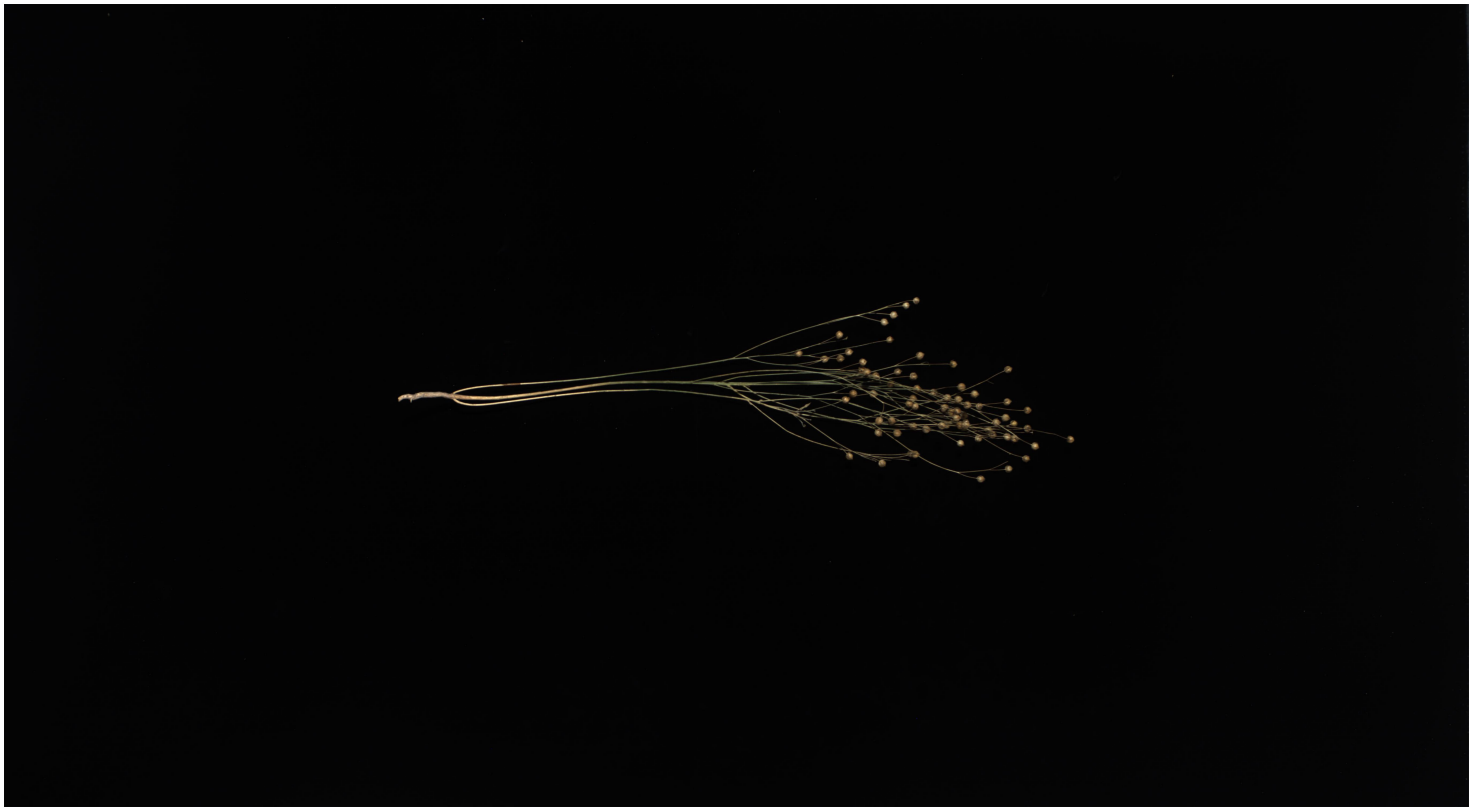
Original image.

**Figure 12:**
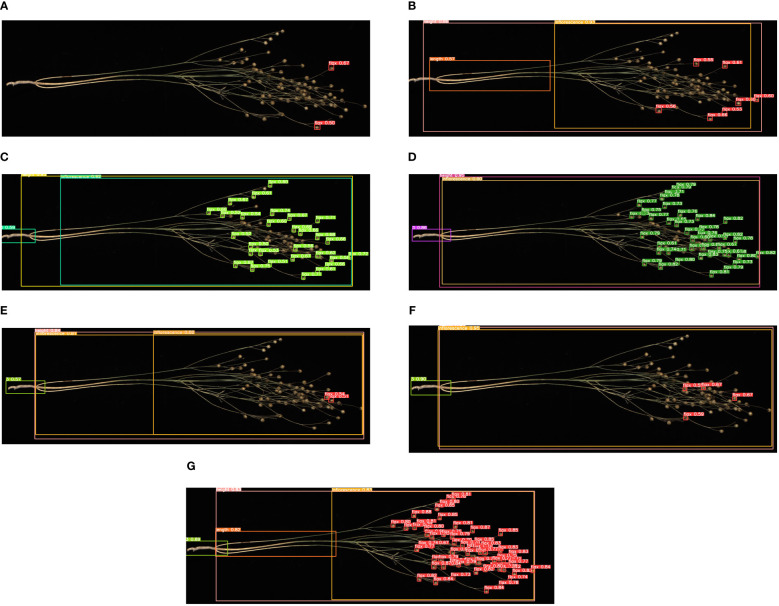
Comparison of recognition of different models: **(A)** YOLOv3-tiny, **(B)** YOLOv5x, **(C)** YOLOv7-tiny, **(D)** YOLOv7x, **(E)** YOLOv8n, **(F)** YOLOv9c, and **(G)** Flax-YOLOv5. The image has been cropped for ease of viewing.

To verify the effectiveness of the model improvement, we selected a flax plant with multiple flax fruits and branches from the test set and tested it using the above model and the Flax-YOLOv5 model; the original image is shown in **Figure 11**, and the comparative results of the recognition by different models are shown in **Figure 12**.

As can be seen in **Figure 12**, the YOLOv3-tiny model has limited recognition ability and can only accurately recognize two flax fruits. Similarly, the YOLOv7-tiny, YOLOv7x, YOLOv8n, and YOLOv9c models were defective in recognizing the main stem length of flax plants, accompanied by a considerable number of missing fruit detection. The improved Flax-YOLOv5 model, in contrast, has better recognition ability and can accurately recognize flax fruits, plant height, main stem length, and number of main stem divisions.

### 3.8 Ablation experiments and analysis

To verify the effectiveness of the improved model Flax-YOLOv5, it is necessary to compare and analyze the models through ablation experiments, and the results of the ablation experiments are shown in **Table 3**.

**Table 3:** Results of ablation experiments.

Model			Number of flax fruits/pieces				Plant height/cm				Main stem length/cm				Number of main stem divisions/pieces			
	BiFormer	SIOU	MAE	HAE	RMSE	R	MAE	HAE	RMSE	R	MAE	HAE	RMSE	R	MAE	HAE	RMSE	R
1	**×**	**×**	1.42	10.00	2.27	99.31	4.02	71.60	14.06	53.57	6.52	51.60	14.04	45.74	0.21	4.00	0.62	76.28
2	**√**	**×**	1.38	8.00	2.21	99.39	3.39	71.30	11.88	65.15	4.48	51.60	10.52	60.93	0.19	3.00	0.50	85.00
3	**×**	**√**	1.37	9.00	2.23	99.19	1.12	3.03	1.37	99.24	6.01	51.60	13.51	45.47	0.17	1.00	0.41	89.67
4	**√**	**√**	1.37	7.00	2.13	99.59	0.80	2.47	1.05	99.53	0.91	2.24	1.12	99.05	0.12	1.00	0.35	92.82

MAE, mean absolute error; HAE, maximum absolute error; RMSE, root mean square error; R, correlation coefficient.

As can be seen in **Table 3**, the correlation coefficients of flax fruits with plant height, main stem length, and number of main stem divisions in Model 2 are higher than the values of Model 1. This observation emphasizes the advantages of the BiFormer network in extracting the target features, which improves the performance of the network in the plant detection task. Model 3 plant height correlation coefficients were significantly higher than those of Model 2 by 34.09 percentage points, which indicates that the integration of SIoU significantly enhanced the model fitting ability, which led to an overall improvement in the accuracy of the model recognition framework.

## 4 Application

To facilitate researchers in selecting flax varieties, it is simple to obtain key phenotypic indicators such as the number of fruits, plant height, main stem length, and the number of main stem divisions of flax plants. Using the improved Flax-YOLOv5 model, the statistical software for flax plant phenotypic data was elaborately designed and developed. This software system is based on PyQt5 technology, which ensures its robustness and scalability. Deployment was effectively accomplished using the PyInstaller toolkit.

The software has a variety of features that greatly assist in phenotypic data analysis. Specifically, users can upload photos and videos and turn on the camera for real-time recognition. By entering data, the software automatically recognizes each organ of the flax plant and provides comprehensive statistics on its phenotypic data. This comprehensive approach ensures accurate and efficient data collection, which is essential for accurate flax variety selection and subsequent breeding programs.

## 5 Conclusion

The acquisition of flax plant phenotype data is the cornerstone of flax breeding. The traditional method is manual technical testing, which is not only time-consuming but also expensive. Therefore, we propose a Flax-YOLOV5 model specifically designed to obtain Flax phenotypic data. The experimental results show that in the verification set, mAP@0.5 is 99.29%. In the test set, the correlation analysis between the predicted value of the model and the key phenotypic traits (fruit number, plant height, main stem length, and main stem number) generated 99.59%, 99.53%, 99.05%, and 92.82%, respectively, and their MAEs were 1.37 pieces, 0.80 cm, 0.91 cm, and 0.12 pieces, respectively, all of which were within the acceptable range. These results show that our method can accurately capture the phenotypic data of flax plants, which provides convenience for the selection of flax varieties. On this basis, a PC-based flax phenotype data collection platform was designed and developed. The platform can efficiently collect key phenotypic traits such as fruit number, plant height, main stem length, and main stem number. This practical application highlights the practicability and effectiveness of our proposed method in supporting flax plant breeding, improves the efficiency of flax plant phenotype data acquisition, and greatly reduces the cost of data acquisition, which provides a solid foundation for flax breeding to become digital. In future research, for plants with complex branches and a large number of fruits, the recognition rate should be further improved, the recognition effect of the number of main stems should be more accurate, and the model parameters should be reduced. At present, the statistics of the secondary branches of the primary branches of flax plants are difficult, and we will further study and solve the problems.

## Data availability statement

The raw data supporting the conclusions of this article will be made available by the authors, without undue reservation.

## Author contributions

KS: Conceptualization, Data curation, Investigation, Methodology, Software, Validation, Visualization, Writing – original draft. CL: Formal Analysis, Funding acquisition, Investigation, Methodology, Project administration, Resources, Supervision, Writing – review & editing. JH: Methodology, Resources, Supervision, Writing – review & editing. JZ: Data curation, Resources, Supervision, Validation, Writing – review & editing. YQ: Data curation, Resources, Supervision, Writing – review & editing.
